# *Cercis*: A Non-polyploid Genomic Relic Within the Generally Polyploid Legume Family

**DOI:** 10.3389/fpls.2019.00345

**Published:** 2019-04-11

**Authors:** Jacob S. Stai, Akshay Yadav, Carole Sinou, Anne Bruneau, Jeff J. Doyle, David Fernández-Baca, Steven B. Cannon

**Affiliations:** ^1^Interdepartmental Genetics and Genomics Graduate Program, Iowa State University, Ames, IA, United States; ^2^Bioinformatics and Computational Biology Graduate Program, Iowa State University, Ames, IA, United States; ^3^Institut de Recherche en Biologie Végétale, Département de Sciences Biologiques, Université de Montréal, Montreal, QC, Canada; ^4^School of Integrative Plant Science, Plant Breeding & Genetics and Plant Biology Sections, Cornell University, Ithaca, NY, United States; ^5^Department of Computer Science, Iowa State University, Ames, IA, United States; ^6^Corn Insects and Crop Genetics Research Unit, US Department of Agriculture–Agricultural Research Service, Ames, IA, United States

**Keywords:** Cercis, polyploidy, legume family, chromosome evolution, whole-genome duplication, ancestral genome

## Abstract

Based on evolutionary, phylogenomic, and synteny analyses of genome sequences for more than a dozen diverse legume species as well as analysis of chromosome counts across the legume family, we conclude that the genus *Cercis* provides a plausible model for an early evolutionary form of the legume genome. The small *Cercis* genus is in the earliest-diverging clade in the earliest-diverging legume subfamily (Cercidoideae). The *Cercis* genome is physically small, and has accumulated mutations at an unusually slow rate compared to other legumes. Chromosome counts across 477 legume genera, combined with phylogenetic reconstructions and histories of whole-genome duplications, suggest that the legume progenitor had 7 chromosomes – as does *Cercis*. We propose a model in which a legume progenitor, with 7 chromosomes, diversified into species that would become the Cercidoideae and the remaining legume subfamilies; then speciation in the Cercidoideae gave rise to the progenitor of the *Cercis* genus. There is evidence for a genome duplication in the remaining Cercidoideae, which is likely due to allotetraploidy involving hybridization between a *Cercis* progenitor and a second diploid species that existed at the time of the polyploidy event. Outside the Cercidoideae, a set of probably independent whole-genome duplications gave rise to the five other legume subfamilies, at least four of which have predominant counts of 12–14 chromosomes among their early-diverging taxa. An earlier study concluded that independent duplications occurred in the Caesalpinioideae, Detarioideae, and Papilionoideae. We conclude that Cercis may be unique among legumes in lacking evidence of polyploidy, a process that has shaped the genomes of all other legumes thus far investigated.

## Introduction

The legume family, Leguminosae, with approximately 20,000 species, is the third most diverse plant family, after Orchidaceae and Asteraceae (Legume Phylogeny Working Group et al., 2017). The family underwent a rapid radiation shortly after its origin ∼59–64 million years ago (Mya) (Lavin et al., 2005; Bruneau et al., 2008), giving rise to six lineages that have recently been recognized as subfamilies by the international legume systematics community (Legume Phylogeny Working Group et al., 2017). Among those subfamilies, four of them (Papilionoideae, Caesalpinioideae, Detarioideae, Cercidoideae) contain the vast majority of genera and species, while Dialioideae contains 17 genera and 84 species, and Duparquetioideae contains a single genus and species. The four larger subfamilies have been shown (Cannon et al., 2015) to each have been affected by early whole-genome duplications (WGDs): at the base of the Papilionoideae and near the origins of the Cercidoideae, Detarioideae, and Caesalpinioideae – though the precise timing of the WGD(s) in the latter three lineages remains uncertain due to low sampling.

In particular, the WGD status and timing within the Cercidoideae has been uncertain: did a WGD predate the earliest divergences in the family, or did it occur later? Cannon et al. (2015) reported a WGD signal for *Bauhinia tomentosa*, based on comparisons of divergence times of duplicated genes and orthologs based on synonymous substitution distributions (*K*_s_ peaks for duplication and speciation) from transcriptome sequence – but no WGD peak was evident for *Cercis canadensis*. This result was inconclusive, however: lack of a WGD peak could have been due to sequence loss or non-recovery for that genus. The genus *Cercis* is sister to the remainder of the Cercidoideae genera (Lewis et al., 2005; Sinou et al., 2009; Wang et al., 2018); we therefore address the question of whether *Cercis* was affected by an early WGD or whether the WGD occurred later in the evolution of the subfamily.

The legumes fall within the Fabidae (rosid 1) clade (Angiosperm Phylogeny Group et al., 2016), and thus were affected by the gamma triplication event that occurred around the time of the origin of the core eudicots, approximately 120 Mya (Jiao et al., 2012). Species such as *Phaseolus* (bean; papilionoid) or *Desmanthus* (bundleflower; caesalpinioid) show evidence of old but independent duplications within the legume family (Cannon et al., 2015). Finding one or more early-diverging legume species without WGD would be of interest because such species could provide important clues to both the structure of the ancestral legume genome and the evolution of species and genomes across this large family.

In the present study, we investigate a new set of genome sequences from the Cercidoideae, Caesalpinioideae, and Papilionoideae, as well as extensive chromosome count data from across the legumes. We also describe results from targeted sequencing of selected genes within the Cercidoideae, to clarify the timing and nature of WGDs affecting the legumes. We present evidence supporting lack of a WGD in the genus *Cercis*, and hypothesize an allotetraploidy event affecting the remainder of the Cercidoideae subfamily.

## Materials and Methods

### Gene Family Construction, *K*_s_ Analysis, and Phylogeny Calculation

Gene families include proteomes (complete sets of translated coding sequences – one representative transcript per gene) from fifteen legume species, and five non-legume species – which were used for phylogenetic rooting and evolutionary context. Species and sources are indicated in Table 1. We used a custom gene family construction method in order to best capture some challenging features of the phylogeny. Gene family features to account for include early WGDs affecting species in the family – but we wished to avoid an older genome triplication, occurring early in angiosperm evolution. Therefore, we used a combination of homology filtering based on per-species synonymous site changes, comparison with outgroup species, Markov clustering, and progressive refinements of family hidden Markov models (HMMs). The gene families are available at https://legumeinfo.org/data/public/Gene_families/legume.genefa m.fam1.M65K/ and associated methods and scripts are available at https://github.com/LegumeFederation/legfed_gene_families although the resources at those locations are focused on papilionoid species rather than on the non-papilionoid species examined in this paper. The same gene families above were used in the analysis in this paper, but with several papilionoid species removed and five other species added (via HMM-search and HMM alignment of the other species to the gene-family HMMs), as shown in Table 1. Resources for these gene families are available in Supplementary Materials: Supplementary Data Sheet S1 (full alignments), Supplementary Data Sheet S2 (trimmed alignments), Supplementary Data Sheet S3 (maximum likelihood trees), and Supplementary Data Sheet S4 (maximum likelihood trees, with same-species terminal pairs reduced to a single representative).

**Table 1:** Genome and annotation sources and versions.

Species	Genotype	Assembly	Annot.	Citation	Source
*Arachis duranensis*	V14167	1	1	Bertioli et al., 2016	PeanutBase
*Arachis ipaensis*	K30076	1	1	Bertioli et al., 2016	PeanutBase
*Cajanus cajan*	ICPL87119	1	1	Varshney et al., 2012	LegumeInfo
*Glycine max*	Williams 82	2	1	Schmutz et al., 2010	Phytozome
*Phaseolus vulgaris*	G19833	2	1	Schmutz et al., 2014	Phytozome
*Vigna radiata*	VC1973A	6	1	Kang et al., 2014	LegumeInfo
*Lotus japonicus*	MG20	3	1	Sato et al., 2008	Phytozome
*Medicago truncatula*	A17_HM341	4	2	Tang et al., 2014	Phytozome
*Cicer arietinum*	Frontier	1	1	Varshney et al., 2013	LegumeInfo
*Nissolia schottii*		1	1	Griesmann et al., 2018	GigaDB
*Mimosa pudica*		1	1	Griesmann et al., 2018	GigaDB
*Chamaecrista fasciculata*		1	1	Griesmann et al., 2018	GigaDB
*Bauhinia tomentosa*		1	1	Cannon et al., 2015	GigaDB
*Cercis canadensis*		1	1	Griesmann et al., 2018	GigaDB
*Prunus persica*	Lovell	2	2.1	International Peach Genome Initiative[IPGI], 2013	Phytozome
*Cucumis sativus*		1	1	Phytozome 12, 2018	Phytozome
*Vitis vinifera*	PN40024	12X	12X	Jaillon et al., 2007	Phytozome
*Arabidopsis thaliana*	Col-0	TAIR10	TAIR10	Berardini et al., 2015	Phytozome
*Solanum lycopersicum*	LA1589	ITAG2.4	ITAG2.4	The Tomato Genome Consortium, 2012	Phytozome


Gene families were generated as follows. All-by-all comparisons of protein sequences for all species were calculated using BLAST (Camacho et al., 2009). Matches were filtered to the top two matches per query, with at least 50% query coverage and 60% identity. For the resulting gene pairs, in-frame nucleotide alignments of coding sequences were calculated, which were used, in turn, to calculate synonymous (*K*_s_) counts per gene pair, using the PAML package (Yang, 2007), with the Nei and Gojobori (1986) method for estimating the numbers of synonymous nucleotide substitutions. The calculation process was driven using the synonymous_calc.py wrapper script (Tang and Chapman, 2018), which additionally uses the packages biopython (Cock et al., 2009), ClustalW2 (Larkin et al., 2007), and PAL2NAL (Suyama et al., 2006). For each species pair, histograms of *K*_s_ frequencies were used as the basis for choosing per-species *K*_s_ cutoffs for that species pair in the legumes. For most species pairs, the selected peak corresponded with the papilionoid duplication (*K*_s_ average of 0.6, varying between 0.45 and 0.8; Supplementary Table S1). For comparisons between papilionoid species and the four non-papilionoid legume species (*Mimosa pudica, Chamaecrista fasciculata, B. tomentosa*, and *C. canadensis*), the selected peak corresponded to the speciation divergence between the pair of species. To accommodate variation in *K*_s_ values, the cutoff for each species pair was generally set at 1.5 times the modal *K*_s_ value (*K*_s_ peak). The set of gene pairs was filtered to remove all pairs with *K*_s_ values greater than the per-species-pair *K*_s_ cutoff. The resulting set of filtered pairs was used for Markov clustering, implemented in the mcl program (Enright et al., 2002), with inflation parameter 1.2, and relative score values (transformed from *K*_s_ values) indicated with the -abc flag. Sequence alignments were then generated for all gene families using MUSCLE (Edgar, 2004). Hidden Markov models (HMMs) were calculated from the alignments using the hmmer package (Mistry et al., 2013), and sequences in each family were realigned to the family that those sequences were assigned to, in order to determine HMM bitscores and calculate a median alignment score for each family. Families were then evaluated for outliers: sequences scoring less than 40% of the median HMM bitscore for the family were removed. The HMMs were then recalculated for each family (without the low-scoring outliers), and were used as targets for HMM search of all sequences in the proteome sets – including those omitted during the initial *K*_s_ filtering. Again, sequences scoring less than 40% of the median HMM bitscore for the family were removed. These HMM alignments were then used for calculating phylogenetic trees, after trimming non-aligning characters (characters outside the HMM match states). Phylogenies were calculated using RAxML (Stamatakis et al., 2008), with model PROTGAMMAAUTO, and rooted using the closest available outgroup species.

### Calculation of *K*_s_ Values and Modal *K*_s_ Peaks

Synonymous-site differences (*K*_s_) were calculated by two methods: first, based on gene-pairs derived from the top two matches of genes between or within species, based on blastp sequence searches; and second, based on gene-pairs derived from genomic synteny comparisons and coding-sequence coordinates, provided to the CoGe SynMap service at https://genomevolution.org/coge/ (Haug-Baltzell et al., 2017). In the former case (calculated on top blastp matches), *K*_s_ values were calculated using PAML, driven by synonymous_calc.py, by Haibao Tang, available at https://github.com/tanghaibao/bio-pipeline. From the PAML output, the Nei-Gojobori *K*_s_ value was used (Nei and Gojobori, 1986). For both approaches (BLAST-based and synteny gene-pair-based), *K*_s_ histograms were calculated after filtering for *K*_s_ values between 0 and 2. The *K*_s_ values and plots are available in Supplementary Table S1.

### Inference of Consensus Branch Lengths From *K*_s_ Peaks

**FIGURE 1:**
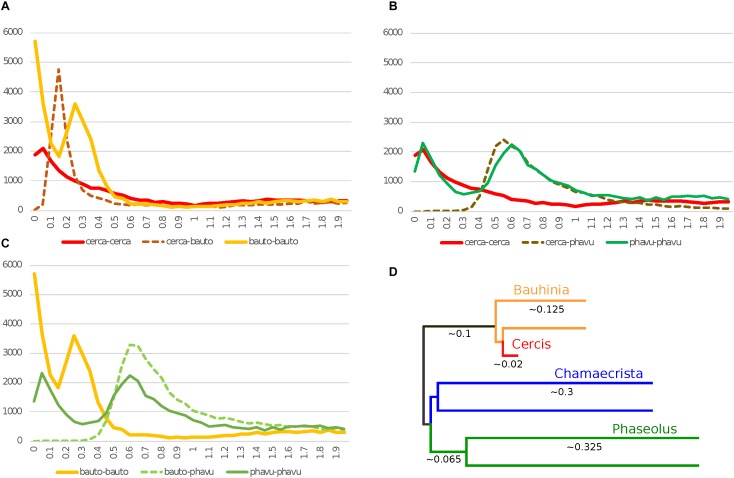
Histograms of *K*_s_ values for top gene-pair comparisons for *Cercis canadensis* (“cerca”), *Bauhinia tomentosa* (“bauto”), and *Phaseolus vulgaris* (“phavu”). In *K*_s_ plots **(A*–*C)**, solid lines are for self-comparisons (e.g., for *Cercis* gene-pairs), and dotted lines are for between-species comparisons (e.g., between *Cercis* and *Bauhinia*). The schematic tree in panel **D** is an idealized distance tree in which each OTU represents an “average” gene: either a single copy in *Cercis*, or each of two homoeologs created by unique WGD events in the remaining taxa. Branch lengths are calculated from pairwise modal *K*_s_ values in panels **A*–*C**.

To infer branch lengths for an idealized gene tree from these *K*_s_ peak values (Figure 1D), modal *K*_s_ peak values were read from *K*_s_ histograms, with values representing WGD events for a species compared with itself (e.g., in *Phaseolus* with respect to the papilionoid WGD) or orthologous gene separations between species (e.g., between *Phaseolus* and *Cercis*). The modal *K*_s_ values were then used to algebraically calculate branch lengths along a gene tree with known species topology and hypothesized duplication history, for the selected species. In these calculations, each branch segment is a variable to be solved, given the observed distances between each terminal (e.g., 0.55 for the phylogenetic path between *Phaseolus* and *Cercis*). Because the internal branch lengths are not uniquely determinable from the observed *K*_s_ path-lengths, several branch lengths were set at 0.01 (based on very short branch lengths observed in both gene trees and species trees): branches subtending the *Chamaecrista* WGD, the papilionoid/caesalpinioid clade, and the *Cercis–Bauhinia* 2 clade. Then, a PHYLIP-format (Felsenstein, 1980) gene tree was manually generated for the represented species, using branch length values from the algebraic calculations.

### Methods for Mining for Tree Topologies

To test the order of phylogenetic events, gene trees were evaluated for 14,709 legume gene family trees that contain *Cercis* and/or *Bauhinia* sequences. Python scripts^1^ that use the functions from the ETE Toolkit (Huerta-Cepas et al., 2010, 2016) were used to read and analyze the legume gene family trees using the species overlap method (Huerta-Cepas et al., 2007). The species overlap method labels an internal node in a given rooted tree as D (duplication event) or S (speciation event) based on whether there are common species between both partitions corresponding to the two subsequent children nodes. Species-overlap tests were run for trees in which same-species terminal pairs were collapsed (when both branch lengths were less than 0.01), to control for local private gene duplications (Supplementary Data Sheet S4).

## Results

### *K*_s_ Peaks From Self-Comparisons of Coding Sequence

Within- and between-species comparisons of rates of synonymous-site changes per synonymous site were evaluated by Cannon et al. (2015) for 20 diverse legume species – including representatives from each of the four largest legume subfamilies. These showed *K*_s_ peaks of around 0.3–0.6 in all species except *Cercis*, where only a much older peak of ∼1.5 was seen. Because that work was based on transcriptome sequence for most species, there was some question whether the absence of the peak in *Cercis* might be due to poor sequence quality or sequence non-recovery (although the transcriptome assembly statistics were generally in the same range as for the other species). Recent availability of genome sequences for *C. canadensis, C. fasciculata, M. pudica*, and *Nissolia schottii*, from Griesmann et al. (2018), provides an opportunity to test *K*_s_ and other results with greater rigor. *Chamaecrista* and *Mimosa* fall within the Caesalpinioideae subfamily, and *Nissolia* is in the Papilionoideae subfamily, within the dalbergioid clade, along with peanut (*Arachis*). For *K*_s_ analysis in this study, we focus particularly on *Cercis, Bauhinia* (as representatives of the Cercidoideae), *Chamaecrista* (as a representative from the Caesalpinioideae), and *Phaseolus* (as a representative of the Papilionoideae), to investigate evidence for the presence and timing of possible WGDs in these lineages. We include *Phaseolus* to provide an example of a species with high-quality genome sequence and a well-studied, early WGD.

*K*_s_ results from genes predicted in the *C. canadensis* (“cerca”) and *C. fasciculata* (“chafa”) genome assemblies are shown in Figure 1, along with genes from *Phaseolus vulgaris* (“phavu”) and from *B. tomentosa* (“bauto”; transcriptome-derived). The *K*_s_ values were determined both for top BLAST-based gene-pairs between species and within species (e.g., top pairs within *Cercis*). Underlying data for the histograms is available in Supplementary Table S1.

**FIGURE 2:**
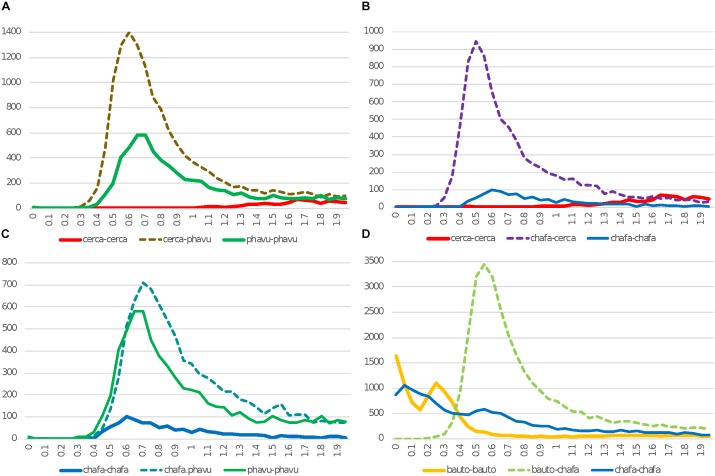
Histograms of *K*_s_ values for synteny-based comparisons for *C. canadensis* (“cerca”), *Chamaecrista fasciculata* (“chafa”), *P. vulgaris* (“phavu”), and *B. tomentosa* (“bauto”). In *K*_s_ plots, solid lines are for self-comparisons (e.g., for *Cercis* gene-pairs), and dotted lines are for between-species comparisons (e.g., between *Cercis* and *Phaseolus*). This Figure differs from Figure 1 both in species selection and in method for selecting gene pairs: in Figure 1, *K*_s_ values are calculated for all top gene pairs, and in panels **A–C**, *K*_s_ values are calculated for gene-pairs from synteny features identified from genomic comparisons (panel **D** is an exception: the *K*_s_ values are calculated from all top gene pairs, because only transcriptomic sequence is available for *Bauhinia*). The effect of using synteny-based gene pairs for calculating *K*_s_ is apparent in the *Chamaecrista* self-comparison plots (chafa*–*chafa; blue) in panel **B** or **C** (syntenic-based) vs. panel **D** (gene-pair based): in the gene-pair based figures in D, the WGD peak is still evident at ∼0.55–0.6, but the signal from more recent gene pairs are also apparent – presumably, as a result of independent, local gene duplications within *Chamaecrista*.

There is a clear *K*_s_ peak for *Cercis–Bauhinia* at 0.15 and a peak for *Bauhinia* compared with itself at 0.25 (Figure 1A,C). Although there are some duplications near 0 in *Cercis* compared with itself, there is no older *Cercis–Cercis* peak as the prominent peak seen in *Bauhinia–Bauhinia* at 0.25. The duplications near 0 in the *Cercis–Cercis* plot are likely due to local gene duplications (as also seen, for example, in the *Phaseolus–Phaseolus* self-comparison in Figure 1A vs 1B), as this signature of recent duplications is absent in the synteny-derived *K*_s_ plots in Figure 2.

We find the expected strong WGD peak within *Phaseolus* and also for *Phaseolus–Cercis* (at 0.6 and 0.55), respectively, but again, no older peak within *Cercis* compared with itself (Figure 1B). The fact that the *Phaseolus–Phaseolus* modal *K*_s_ peak is greater than the *Phaseolus–Cercis* peak suggests a much greater rate of mutation accumulation in *Phaseolus* and its progenitors in Papilionoideae than in *Cercis* and its progenitors in Cercidoideae (Cui et al., 2006; Schmutz et al., 2014).

In Figure 1C, there is a speciation peak for *Phaseolus–Bauhinia* that is similar to *Phaseolus–Cercis* with the exception that the *Phaseolus–Bauhinia* peak appears slightly “older” than for *Phaseolus–Cercis* (0.6 vs. 0.55), suggesting more rapid rate of mutation accumulation in *Bauhinia* than in *Cercis*.

Figure 1D shows an inferred consensus gene tree, with branch lengths calculated (with approximation) from *K*_s_ plots in Figure 1, 2 (as described in Methods).

In Figure 2A–C, *K*_s_ values are derived from gene-pairs within synteny blocks derived from genome comparisons. A major effect of this strategy is to exclude local gene duplications – and to reduce other paralogous matches that can show up as recent duplications – for example, in matches among many members of a recently expanded gene family. This reduction in recent- and locally derived paralogs is evident in *K*_s_ counts near zero for “young” (small) *K*_s_ values. The sloping *K*_s_ histogram seen in Figure 1 for *Cercis–Cercis* is entirely absent in Figure 2. The modal *K*_s_ “peak” for *Cercis*, if there is any, is in the range of 1.5–2 – contrasting with the *Cercis–Phaseolus, Cercis–Chamaecrista*, and *Chamaecrista–Phaseolus* peaks of 0.6, 0.5, and 0.7, respectively – indicating that any *Cercis* WGD peak in this data would well predate the legume origin.

Also noteworthy in Figure 2 is the low modal *K*_s_ peak for *Chamaecrista–Chamaecrista* (amplitude of 101, compared with 581 for *Phaseolus–Phaseolus*). This difference in numbers of paralogous duplicated genes could be due to higher rates of gene loss from *Chamaecrista* following WGD early in the Caesalpinioideae. The strong *K*_s_ peaks in the orthologous *Chamaecrista* – *Cercis* comparison and the *Phaseolus* – *Cercis* comparison suggest that there is nothing systematically wrong with the *Chamaecrista* gene models. Rather, it appears that *Chamaecrista* is more fully “diploidized,” with a higher proportion of duplicated genes having reduced to single copies, providing a sufficient basis for discovering correspondences with other species, but erasing much of the WGD signature in a *Chamaecrista* self-comparison. Similar diploidization and interspersed gene losses have been reported in *Medicago truncatula* (Young et al., 2011).

### Genomic Synteny Analysis

Given the draft genomic sequence assembly for *Cercis*, it is possible to make synteny comparisons with other legume genome assemblies, as well as assemblies of near outgroups to the legumes. In a synteny comparison of two genomes, a WGD present in one of the genomes and absent in the other should be apparent in a genomic dotplot through the following pattern: starting from a given genomic region in the non-duplicated genome and tracing through the dotplot, one should find matches to two regions in the genome with the WGD; and starting from a given genomic region in the duplicated genome and tracing through the dotplot in the other axis, one should find matches to a single region in the genome that lacks the WGD. This can be described in terms of “synteny depth:” the depth of the duplicated genome should be twice that of the non-duplicate genome.

Because the *Cercis* assembly is still highly fragmented (N50 of 421 kb), synteny depth is difficult to assess visually, but it can be measured computationally. The quota-alignment package (Tang et al., 2011) identifies synteny blocks between two genomes, attempting to match a specified pair of synteny depths or “quotas.” For example, if genome B has a WGD that A lacks, then the quota for B relative to A would be 2:1. If the quota is mis-specified as 1:1, then a poor coverage score will result for the duplicated genome, because many potential blocks in genome B will be missed. We also note that in the quota-alignment package, in a genome self-comparison, the trivial self-match is suppressed, so the expected quota for a genome with a single WGD, compared with itself, would be 1:1 rather than 2:2.

We used the quota-alignment package to test a range of quotas for all comparisons among *Cercis, Phaseolus*, and *Prunus*. We also provide the corresponding plots and textual results in Supplementary Data Sheets S8, S9. There is no evidence for a duplication in *Prunus* since the angiosperm whole-genome triplication (WGT) (Jiao et al., 2012; The International Peach Genome Initiative et al., 2013), and there is a known WGD in *Phaseolus* at around 50 Mya (Schmutz et al., 2014; Cannon et al., 2015), so these should serve as useful comparisons relative to *Cercis*. For *Prunus–Phaseolus*, a quota of 1:1 gives *Phaseolus* coverage of only 63.8% (Table 2) vs. 96% for *Prunus*, indicating that less than two-thirds of the *Phaseolus* genome has synteny coverage for the identified gene pairs. A quota of 1:2 for *Prunus–Phaseolus* is much better, at 97.4 and 96.8% coverage, respectively. For *Prunus–Cercis*, a quota of 1:1 gives acceptable coverage of 93.4 and 95.2%, respectively; a quota of 1:2 improves the coverage by only about 2% (Table 2). For *Phaseolus–Cercis*, the best quota is 2:1, with coverages of 93.3 and 94.7%, respectively. For the self-comparisons for each species, there is notable improvement going from 1:1 to 2:2 (Table 2). This is likely due to the ancient angiosperm triploidization (Jiao et al., 2012), which generated three genome copies; the expected number of synteny blocks from any region would then be two (ignoring the trivial self-match).

**Table 2:** Synteny coverage for comparisons between the genomes of *Cercis canadensis, Phaseolus vulgaris*, and *Prunus persica*, at selected synteny “quotas” (expected coverage depths).

Quotas	*X*	*Y*	*K*_s_ peak	Comments
	**Cercis**	**Cercis**		

q1-1	87.1	87.8	1.74	OK
q2-2	99.9	99.9		BEST

	**Phaseolus**	**Cercis**		

q1-1	**61.9**	**94.1**	**0.62**	At q1:1, Phaseolus coverage is too low
q2-1	93.3	94.7		BEST

	**Prunus**	**Cercis**		

q1-1	93.4	95.2	0.92	OK
q1-2	94.1	97.8		little improvement over q1:1
q2-2	99.2	98.6		BEST

	**Phaseolus**	**Phaseolus**		

q1-1	91.7	92.0	0.70	OK
q2-2	98.9	98.9		BEST

	**Prunus**	**Phaseolus**		

q1-1	**96.0**	**63.8**	**1.16**	At q1:1, Phaseolus coverage is too low
q1-2	97.4	96.8		BEST

	**Prunus**	**Prunus**		

q1-1	84.7	84.2	1.40	OK
q2-2	99.6	99.2		BEST


*For the comparison between *Prunus* and *Phaseolus* (with known WGD histories), the best quota choice is 1:2, corresponding with two synteny blocks in *Phaseolus* for one in *Prunus*. Similarly, for the comparison between *Cercis* and *Phaseolus*, the best quota choice is 1:2, corresponding with two synteny blocks in *Phaseolus* for one in *Cercis*; and for the comparison between *Cercis* and *Prunus*, the best quota choice is 1:1, suggesting that neither genome has a recent WGD in its history. The *K*_*s*_ peak values are consistent with this conclusion – with the *Cercis–Cercis* being of comparable age to *Prunus–Prunus* (and likely dating to the angiosperm whole-genome triplication). Values in bold highlight cases where quota choices are particularly ill-fitting, and therefore informative as inappropriate models of WGD histories.*

The *K*_s_ peak values derived from gene pairs in the synteny analysis (Table 2) are consistent with the synteny depth results – with the *Cercis–Cercis* peak being of comparable age to *Prunus–Prunus* (1.74 and 1.4, respectively), and likely both dating to the angiosperm WGT. In contrast, the peak for *Phaseolus–Phaseolus* is 0.7, consistent with the papilionoid WGD.

Taken together, the synteny and *K*_s_ results from Table 2 indicate that *Cercis* has the same overall WGD depth as *Prunus* and half that of *Phaseolus*, in comparisons among these genomes. In other words, the synteny and *K*_s_ evidence supports lack of a WGD in *Cercis*.

### Phylogenomic Analyses

To determine duplication events in a phylogenetic context, we constructed gene trees for all legume genes, for fifteen diverse legume species: *Glycine max, P. vulgaris, Vigna unguiculata, Lupinus angularis, Arachis ipaensis, N. schottii, Cicer arietinum, M. truncatula, Lotus japonicus, C. fasciculata, M. pudica, B. tomentosa*, and *C. canadensis*. The first nine of these are from the Papilionoideae (representing the millettioid, genistoid, dalbergioid, and IRLC clades). We also included five non-legume outgroups – using one sequence from each, for each family, in order to provide a rooting for the legume sequences: *Arabidopsis thaliana, Prunus persica, Cucumis sativus, Solanum lycopersicum*, and *Vitis vinifera*. For convenience, analyses and figures that use sequences from these species use the following abbreviation form to indicate genus and species: the first three letters of the genus and the first two letters of the species epithet, e.g., “glyma” for *G. max*. Gene families were calculated to span the depth of the legume most-recent common ancestor – i.e., avoiding fragmented gene families that split sequences that have a common proto-legume ancestor, and avoiding over-clustered families that include legume sequences that diverged prior to the legume origin. Our method produced 18,543 such families, but for the present analysis, we analyzed the 14,709 families that contain one or more sequences from *Cercis* and/or *Bauhinia*. The set of 14,709 were used for subsequent phylogenomic analyses (Supplementary Data Sheets S1–S4).

### Informal Observations About Patterns in Trees

**FIGURE 3:**
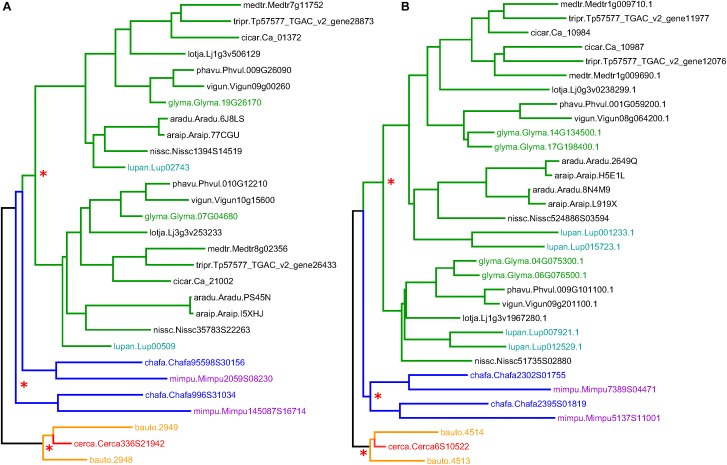
Sample gene trees (for gene families 31DXWY and 2SH9KY; **A** and **B**, respectively), showing clades corresponding to the Cercidoideae (orange and red), Caesalpinioideae (blue and violet), and Papilionoideae (green). Species abbreviations are composed of the first three letters from the genus and the first two letters of the species. Full name correspondences are indicated in the text. Non-legume outgroup sequences are in gray. Red asterisks mark common ancestors of homoeologous sequence pairs. Additional, more recent WGDs within the Papilionoideae are highlighted with colors of the sequence IDs: green for *Glycine max* and turquoise for *Lupinus angustifolius*.

Gene family trees containing *Cercis* and *Bauhinia* sequences were used to investigate the occurrence of WGD in the most recent common ancestor (MRCA) of the *Cercis* and *Bauhinia* lineages. Although the phylogenomic analysis was likely complicated by uncertainties in phylogenetic reconstructions and by sequence losses or non-recovery, there are clear patterns in the results. We repeatedly see topologies congruent with those in two gene families shown in Figure 3 (families 31DXWY and 2SH9KY; names from this set of legume gene families were assigned random “license plate” names of six alphanumeric characters). These gene families each show two *Bauhinia* sequences and one *Cercis* sequence in one clade. Both gene families show duplicated sequences for *Mimosa* and *Chamaecrista* (Caesalpinioideae; although in 3A, these do not resolve to a single clade, which may indicate that the duplication occurred very early in the Caesalpinioideae) in the Papilionoideae, there are paired sequences from most species, highlighting the pre-papilionoid WGD (Cannon et al., 2015). In the Cercidoideae clade, there is a curious feature: the duplication that affects *Bauhinia* predates the *Bauhinia–Cercis* speciation, and produces the expected two homoeologs in *Bauhinia*, but there is only a single *Cercis* sequence. The full collection of gene trees is available in Supplementary Data Sheet S3.

### Summaries of Sequence Counts for All Gene Families (Legume Phylogeny Working Group et al., 2017)

To investigate WGDs in the legumes, we analyzed gene counts across all legume gene families. A summary overview of the phylogenomic analysis is shown in Table 3, which gives counts of gene families (and trees) having the indicated sequence count for each species (Only selected species are shown in Table 3; the counts for all species and all families are given in Supplementary Table S2). These are given for two variants of the trees: first (A) for the full, unmodified trees, and second (B) for trees in which similar (*K*_s_ < 0.2) terminal sequence pairs for a species have been reduced to a single representative, in order to reduce the effect of private, genus-specific WGDs. For example, in Table 3A, the first column (glyma / *G. max*) shows the largest number of trees (6531) having two sequences, and the second largest number of trees (3995) having four or more sequences. A count of four for *G. max* would be expected in a gene family in which no gene loss occurred following the two WGDs in the *Glycine* lineage within the period of legume evolution (Schmutz et al., 2010). In Table 3B, in which terminal same-species pairs have been reduced to a single representative, the largest number of trees (7951) has one sequence, and the second largest number of trees (4217) has two sequences.

**Table 3:** Counts of gene families with the indicated numbers of genes per family.

*count*	glyma	phavu	aradu	Nissc	medtr	tripr	lotja	chafa	mimpu	bauto	cerca
**(A) Counts for original full trees.**										

0	553	826	2264	1425	1001	1252	1873	2558	3859	4066	1557
1	1933	8748	7761	8472	8141	8255	7602	7894	6432	5921	10567
2	6531	3981	3390	3656	3545	3429	3444	3178	2858	2570	1708
3	1697	716	752	681	984	957	1138	591	846	1130	437
≥ 4	3995	438	542	475	1038	816	652	488	714	1022	440
≥2/ = 1	632%	59%	60%	57%	68%	63%	69%	54%	69%	80%	24%
**(B) Counts for trees with terminal recent pairs per species are reduced to a single representative.**										

0	553	826	2265	1427	1003	1254	1873	2559	3860	4067	1558
1	7951	9034	7907	8815	8806	8878	9018	8353	7934	7475	10988
2	4217	3911	3396	3621	3443	3285	3066	2970	2160	2362	1564
3	1163	616	707	545	798	791	534	484	430	546	342
≥ 4	825	322	434	301	659	501	218	343	325	259	257
≥2/ = 1	78%	54%	57%	51%	56%	52%	42%	45%	37%	42%	20%


*Numbers for a given count (left-hand column) are the numbers of families with counts per species for the given count categories. This Table gives counts for the full gene families, and counts for gene families in which similar (*K*_*s*_ < 0.2) terminal sequence pairs for a species have been reduced to a single representative, in order to reduce the effect of private, genus-specific WGDs. Ratios of counts are given below each table: for a given species, the number of families with one sequence in the family over the number of families with two or more sequences in the family. This ratio provides an indication of possible whole-genome duplications present for that species.*

We propose that an indicator of potential older WGDs for a species is obtained by dividing the number of gene family counts for which a species is represented at least twice in the family by the number of family counts for which a species is represented only once. These ratios are given at the bottom of Tables 3A,B. For species with a WGD within the period of legume evolution, a relatively larger number of families should have two or more sequences. The most dramatic ratio is for *Glycine* (632%; i.e., 6.3 × the naïve expectation) – which has two WGDs in its legume history (pre-papilionoid and a much more recent *Glycine*-specific duplication). For the unreduced trees (1A), all other species have ratios greater than 50% except for *Cercis*, with 24%. For the reduced trees (with collapsed terminal same-species clades), the ratios are somewhat lower for all species: 42–78% for all species except *Cercis*, with 20%. We interpret these results as evidence for WGD in all of the represented legume species except *Cercis*.

### Mining for Tree Topologies Within the Cercidoideae

**FIGURE 4:**
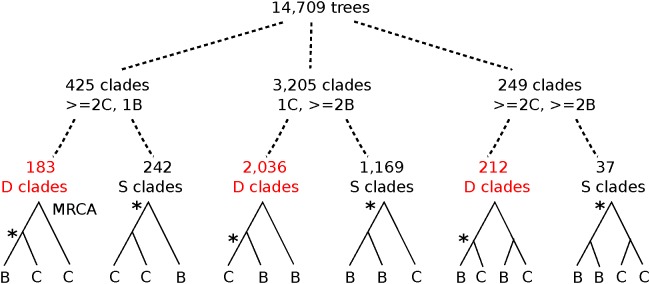
Graphical depiction of tree-mining results for topologies in the Cercidoideae. From 14,709 family trees with *Cercis* and *Bauhinia* sequences, clades with ≥2 *Cercis* and one *Bauhinia* sequence were 7.5 times more common than clades with 1 *Cercis* and ≥2 *Bauhinia* sequences (425 vs. 3205 clades, respectively). Of the latter (more frequent) clade configuration, cases with [**(C,B)**,**B**] are 1.74 times more common than cases with [**(B,B)**,**C**] (2036 vs. 1169 clades, respectively). In the first of these patterns, [**(C,B)**,**B**], the MRCA node of the clade is labeled as a Duplication by the “species overlap” algorithm (see section “Materials and Methods” for description) – meaning that a the MRCA is inferred as due to a gene duplication event rather than a speciation-derived orthology event. Asterisks mark nodes where orthologous genes derive from speciation. Also see Table 4 for counts and percentages.

To infer the relative timing of gene duplications relative to speciations, we mined legume gene phylogenies for topological patterns expected to be produced by these events. Monophyletic groups were detected from a set of 14,709 families containing at least one sequence each from *Cercis* and *Bauhinia* (Figure 4 and Table 4). The MRCA node for each clade containing *Cercis* and *Bauhinia* was labeled either as D (for a duplication event) or S (for a speciation event), based on whether there are common species between both partitions corresponding to the two subsequent children nodes. For example, considering clades with two sequences from each of *Bauhinia* and *Cercis*, [(B,C),(B,C)] would be labeled D while [(B,B),(C,C)] would be labeled S (Figure 4) The species overlap method has been previously used to study evolutionary relationships of human proteins with their respective homologs in other eukaryotes (Huerta-Cepas et al., 2007). We considered three types of monophyletic groups varying by number of *Cercis* and *Bauhinia* sequences: clades containing ≥ 2 *Cercis* and ≥ 2 *Bauhinia* sequences, clades containing exactly 1 *Cercis* and ≥ 2 *Bauhinia* sequences, and finally clades containing exactly 1 *Bauhinia* and ≥ 2 *Cercis* sequences. The proportions of clades out of the total number of clades, for all the three types, that were labeled as D at the MRCA node were also calculated. Species-overlap tests were run on trees in which very recently derived same-species terminal pairs were collapsed (when both branch lengths were less than 0.01), to control for local private gene duplications.

**Table 4:** The types of monophyletic groups containing different numbers of *Cercis* and *Bauhinia* sequences.

# of *Cercis* seqs. in clade	# of *Bauhinia* seqs. in clade	total # of clades detected	# of clades labeled as duplication at MRCA	percent of duplication clades
≥ 2	≥2	249	212	85%
≥2	1	425	183	43%
1	≥2	3205	2036	63%


*For example, there are 425 clades with ≥ 2 *Cercis* sequences and 1 *Bauhinia* sequence. The last column indicates the proportion of clades with a duplication pattern consistent with WGD having occurred prior to the Cercis–Bauhinia speciation, e.g., [B,(B,C) or (C,(B,C)], as opposed to a speciation pattern, e.g., [(B,B),C] or [B,(C,C)].*

There are approximately tenfold more trees with one *Cercis* and two or more *Bauhinia* sequences than with one *Bauhinia* and two or more *Cercis* sequences (Table 4; 425/3205 and 183/2036). We interpret this result (preponderance of the 1 *Cercis*, ≥ 2 Bauhinia pattern) as evidence for WGD in *Bauhinia* but not *Cercis*. Further, of the clades with two or more *Bauhinia* sequences and one *Cercis* sequence, most (63%) of these have *Cercis* nested within the clade: 2036 of the total clade count look like [(B,C),B] rather than [(B,B),C] – the former likely resulting from a duplication of *Bauhinia* prior to speciation, and the latter resulting from speciation followed by duplication of *Bauhinia*. This result might seem nonsensical (duplication predating the *Cercis–Bauhinia* speciation, yet not affecting *Cercis*), but it would be consistent with allopolyploidy – with a *Cercis* progenitor having contributed one of the subgenomes in the allopolyploidy event that gave rise to *Bauhinia* and all other species in the rest of the Cercidoideae clade (elaborated further in the section “Discussion”).

### Gene Duplication Patterns Across Diverse Species in the Cercidoideae

**FIGURE 5:**
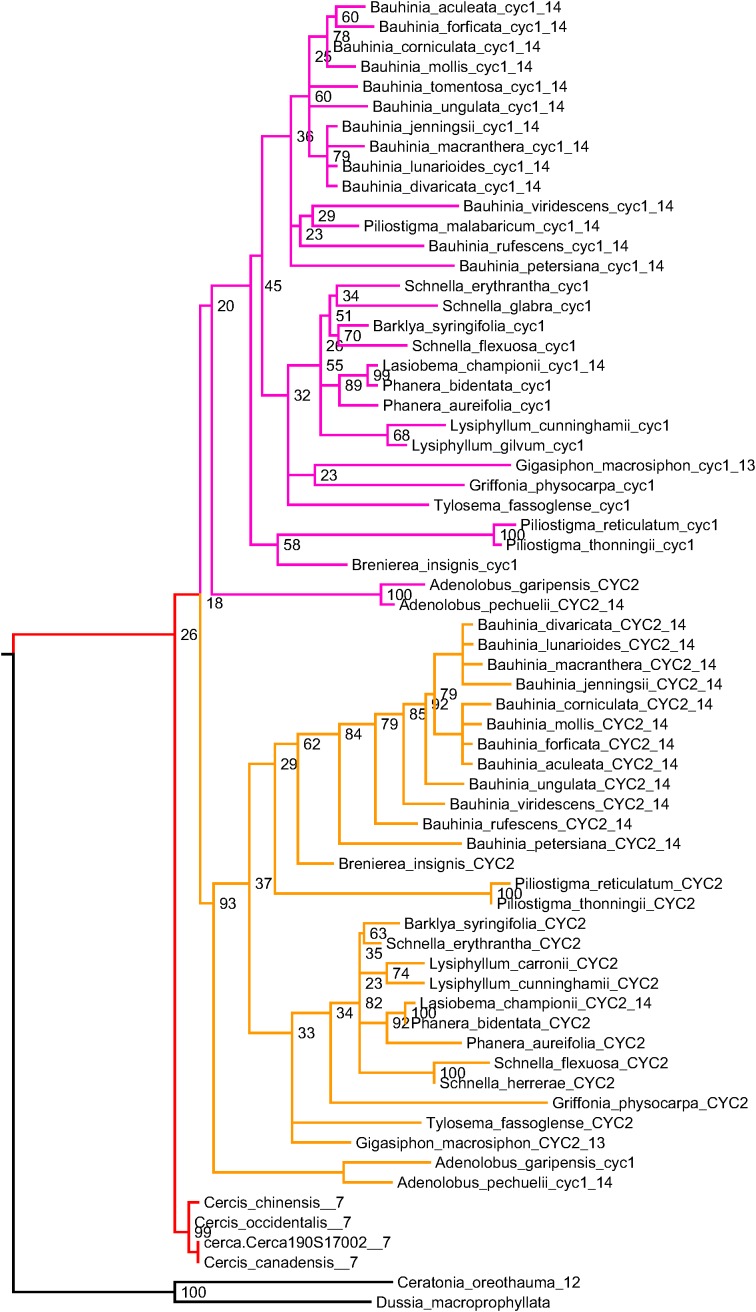
*CYCLOIDEA* gene tree, for species in subfamily Cercidoideae. For all species but *Cercis* (red), there are two gene copies: in the clades labeled “CYC1” (pink) and “CYC2” (orange). Where chromosome counts are available, the haploid count is indicated at the end of the sequence identifier. These values are 7 for the three included *Cercis* species, and 14 for all other species for which counts have been determined within the Cercidoideae, save *Gigasiphon macrosiphon*, which has 13. For *C. canadensis*, one sequence has been amplified using PCR and one sequence (Cerca190S17002) comes from the genomic assembly. One of several possible rootings is shown (with bootstrap support values indicated), based on comparison with *CYCLOIDEA* orthologs from *Ceratonia oreothauma* (carob relative, from the Caesalpinioideae) and *Dussia macroprophyllata* (an early-diverging species from the Papilionoideae).

To determine gene duplication patterns for species in the Cercidoideae, we take advantage of the well-conserved *CYCLOIDEA*-like TCP genes, which have been used both for phylogenetic inference and for studies of evolutionary development in the legumes (Citerne et al., 2003, 2006). Using two sets of degenerate PCR primers that preferentially amplify two classes of *CYCLOIDEA*-like TCP genes in the legumes (Citerne et al., 2003), Sinou and Bruneau (pers. comm.) amplified *CYCLOIDEA*-like genes from 114 species in Cercidoideae. These span all twelve genera in this subfamily. A phylogeny from a subset of these sequences is shown in Figure 5 – with sequences from each genus included but omitting some species from well-represented genera (see Supplementary Data Sheet S5 for the phylip-format phylogeny and SD08 for the sequence data and accessions).

A feature readily apparent in the phylogeny is its division into three clades: one with sequences marked “CYC1” (salmon), one with sequences marked “CYC2” (orange), and one unlabeled (red) (Figure 5). Most species have two representatives in the phylogeny: one in the CYC1 clade and one in the CYC2 clade – except in *Cercis* (three species), for which only one sequence was amplified (or recovered from the genome assembly, in the case of *C. canadensis*). Although the favored topology places *Cercis* sequences sister to sequences from other Cercidoideae, bootstrap support for this relationship is weak. Alternative resolutions thus are not ruled out, including placement of the *Cercis* clade sister to either CYC1 or CYC2. This would be consistent with the pattern observed in the trees in Figure 3, i.e., [(C,B1),B2] – and would be consistent with a model of allopolyploidy (see section “Discussion”).

### Chromosome Counts Across the Legume Phylogeny

Phylogenetic and chromosome count data can be combined in order to explore chromosomal evolution across the legumes. We combined the extensive *matK*-based phylogeny from the LPWG (Legume Phylogeny Working Group et al., 2017), with count data from the Chromosome Counts Database (CCDB version 1.45) (Rice et al., 2015). The CCDB contains 27,947 count reports for legume species, spanning 477 genera. For many genera, there are numerous reports; for example, *Acacia* has 472 reported counts across 152 species. We determined the modal gametic chromosomal count value, “*n*,” for each genus (for example, in *Acacia*, the modal count is *n* = 13, of the 152 species with counts, 71% have *n* = 13). See Supplementary Table S4 for count details. We then displayed these modal counts on the species phylogeny, using one species as the representative for each genus in the phylogeny.

**FIGURE 6:**
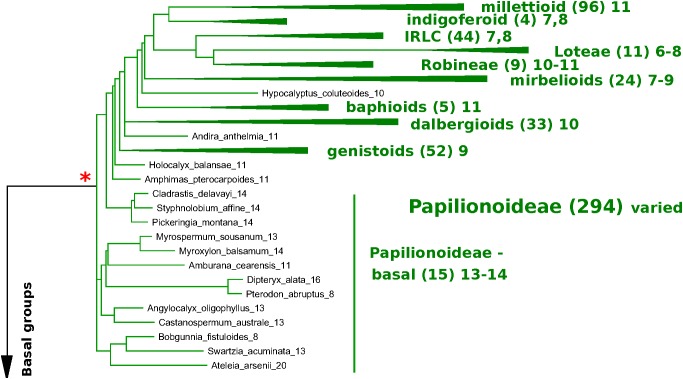
Papilionoid portion of the *matK*-based species phylogeny for representative species in the legumes, with chromosome count data (Figure 6, 7). *matK*-based species phylogeny for representative species in the legumes (derived from Legume Phylogeny Working Group et al., 2017), with chromosome count data. Only species for which chromosome counts are available are shown, with the exception of the Cercidoideae (Figure 7), where additional species are shown for context in that subfamily. Chromosomal counts are given as the mode for the indicated genus, where there are differences in the genus. Some particularly well-represented clades have been collapsed and are represented by a colored triangle. The number of genera with counts is given in parentheses – for example, 96 genera are represented in the triangle representing the millettioid clade (top of Figure 6), and 47 genera are represented in the triangle representing the Mimosoid clade (top of Figure 7). Red asterisks indicate polyploidy events – either known (e.g., Papilionoideae) or hypothesized (e.g., Dialioideae).

**FIGURE 7:**
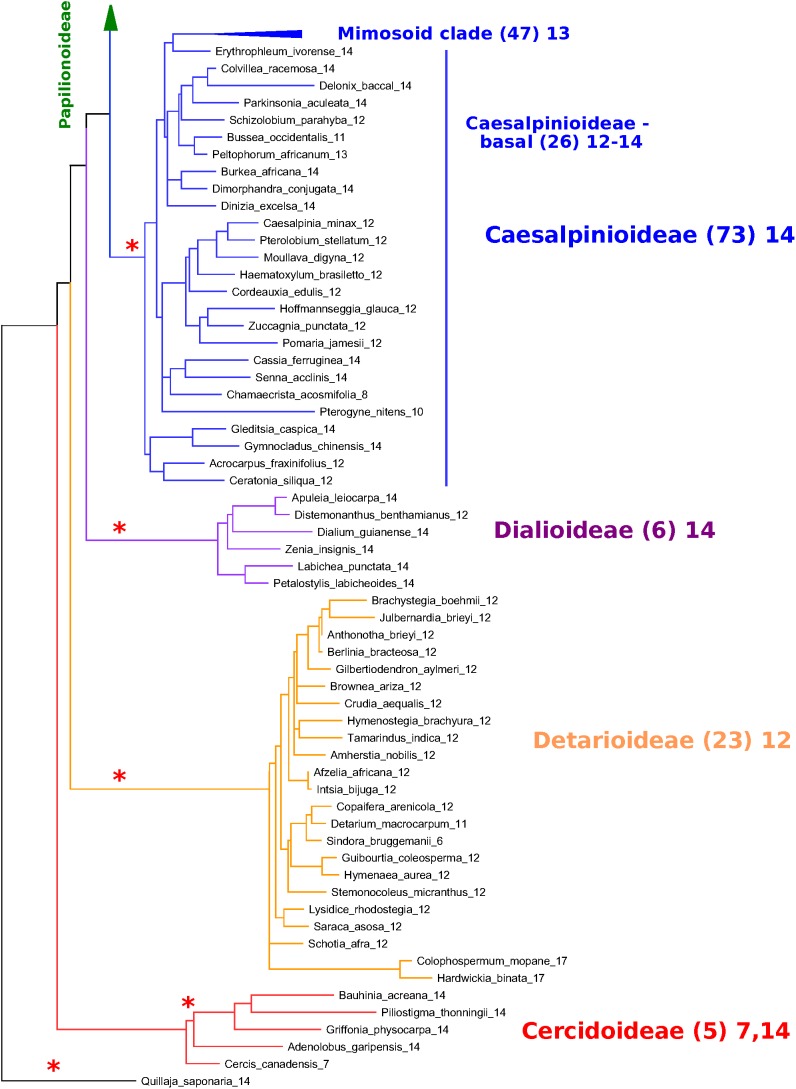
Non-papilionoid portion of the *matK*-based species phylogeny for representative species in the legumes, with chromosome count data. Figure 7 extends Figure 6; see description under Figure 6. The relative placements of the subfamilies are uncertain, with the Cercidoideae and Detarioideae, best considered as a polytomy, given current phylogenetic resolutions (Legume Phylogeny Working Group et al., 2017). ^∗^Indicate polyploidy event – either known (e.g., Papilionoideae) or hypothesized (e.g., Dialioideae).

In Figure 6, 7, a partially collapsed phylogeny has been annotated and summarized for ease of presentation. Count details for each species and genus are given in Supplementary Table S4; an image of the full tree with count data is in Supplementary Data Sheet S6; and the PHYLIP-format tree file is in Supplementary Data Sheet S7. Some particularly well-represented clades have been collapsed; for example, the mimosid clade contains 47 species with chromosomal counts; these have been collapsed in Figure 7, and the overall modal count for that clade is presented as an annotation (the mode for the chromosomal count is *n* = 14 for the mimosoid clade within the Caesalpinioideae). See Table 5 for counts in each clade.

At the subfamily level, the modal chromosome counts are generally unambiguous, with the exception of the Papilionoideae, with a more complex pattern of chromosome counts. The Papilionoideae, being an unusually large subfamily (containing ∼13,800 species in that subfamily and more than 70% of legume species; Cardoso et al., 2012), has been treated in a separate analysis (Ren et al., 2019). However, we note here that the groups sister to the large crown clades of papilionoid species, e.g., *Swartzia, Myroxylon*, and *Cladrastis*, have 13 and 14 as the most frequent counts (Figure 6 and Table 5). The clades of the crown group generally have lower counts: 11 for *Amphimas, Holocalyx, Andira* dispersed along the grade with the genistoid, dalbergioid, and baphioid clades. Among the remaining papilionoid clades (containing the majority of species in the subfamily), chromosome counts are varied, but are generally in the range of 7–11 chromosomes.

The Caesalpinioideae has generally clear count patterns: 14 for the large mimosoid clade and 12–14 for the remaining, early-diverging taxa (Table 5). Across 73 genera with counts in the Caesalpinioideae, 66 have modes at *n* = 12, 13, or 14 (14, 35, 17, respectively – combining “early” and “mimosoid” in Table 5). There are some intriguing exceptions, however; for example, *Calliandra* and *Chamaecrista* and have *n* = 7–8, despite being nested in clades with *n* = 13 or 14 – apparently indicating chromosomal fusions or reductions of some sort; and other genera such as *Neptunia* and *Leucaena*, have *n* = 28 and 52, respectively, suggesting ploidy increases from *n* = 14 and 13.

For the Dialioideae, five of six genera with count data have *n* = 14. For the Detarioideae, 19 of 23 genera with count data have *n* = 12. For the Cercidoideae, four genera (*Bauhinia, Piliostigma, Griffonia, and Adenolobus*) with count data have *n* = 14, and only *Cercis* has *n* = 7. The nearest outgroup species to the legumes may also be informative. *Quillaja saponaria* (Quillajaceae) which shows evidence of a WGD (via transcriptome *K*_s_ data; Cannon et al., 2015), has *n* = 14. Another near outgroup, *Suriana maritima* (Surianaceae), has *n* = 9; its WGD status is not known directly, though it lacks duplication in any of its CYC-like genes (Zhao et al., 2019).

### Genome Sizes in the Cercidoideae

Roberts and Werner (2016) report an average of 2*C* = 0.751 pg for 30 accessions across 9 *Cercis* species. Using the conversion ratio of 1 pg = 978 Mb (Dolezel et al., 2003), this gives a *Cercis* genome size estimate of 1*C* = 0.78 pg ^∗^ (978 Mb / 1 pg) / 2 = 367 Mbp. This compares with reported 1C genome sizes for several *Bauhinia* species: 573 Mbp for *B. purpurea*; 613 Mbp for *B. tomentosa*, and 620 Mbp for *Lysiphyllum hookeri* (formerly *B. hookeri*) (Bennett and Leitch, 2005). These values are ∼1.5 to ∼1.6 times larger than *Cercis* – which is consistent with the *Bauhinia* genomes having doubled relative to *Cercis* (followed by moderate increase in *Cercis* and/or decrease in *Bauhinia* – or a situation of an allopolyploid *Bauhinia* being derived from two genomes of different sizes – one contributed by a *Cercis* progenitor and one presumably now extinct). A size of 381 Mbp for *Cercis* is also small relative to other reported legume genomes; for example, the estimated sizes of *L. japonicus, M. truncatula, P. vulgaris*, and *C. arietinum*, respectively, are 472–597 Mbp, 465–562 Mbp, 587–637, 738–929 (Arumuganathan and Earle, 1991; Sato et al., 2008; Bennett and Leitch, 2011; Varshney et al., 2013; Tang et al., 2014). Indeed, in comparison with genome size reports for 722 legume species and 84 genera from the Kew *C-value* database (Bennett and Leitch, 2012), the *Cercis* estimate of *n* = 367 Mbp would be smaller than all but one other legume genome (*Lablab niger* also has an estimated size of 367 Mbp). For all reported legume genera (taking median value per genus where values are available for multiple species in a genus), the average haploid genome size is 1,424 Mbp and the median is 1,157 Mbp (Supplementary Table S5).

**Table 5:** Counts of genera with indicated haploid (gametic) chromosome numbers, by subfamily or clade.

**Clade∖Count**	6	7	8	9	10	11	**12**	**13**	**14**	15	16	> 16	total	frequent

Papilionoid – derived	4	21	**57**	**36**	**39**	**77**	6	0	5	0	6	27	278	8–11
Papilionoid – grade	0	0	0	0	0	**3**	0	0	0	0	0	0	3	11
Papilionoid – early	0	0	2	0	0	1	0	**4**	**4**	0	1	1	13	13–14
Caesalp – mimosoid	0	0	1	0	0	0	1	**31**	5	0	0	3	41	13
Caesalp – early	0	0	1	0	1	1	**13**	**4**	**12**	0	0	0	32	12–14
Dialidoideae	0	0	0	0	0	0	1	0	**5**	0	0	0	6	14
Detarioideae	1	0	0	0	0	1	**19**	0	0	0	0	2	23	12
Cercidoideae	0	**1**	0	0	0	0	0	0	**4**	0	0	0	5	7,14


*Each cell (except for the count summaries in the last three columns) contains the number of genera with a chromosome count indicated (column), for that clade (row). For example, in the Caesalpinioideae (which includes the mimosoid clade), 31 genera have a chromosome count of 13. (For most genera, all species have the same chromosome count, but where count differences are reported in the literature, the modal value is used for the genus). For each clade, the most frequent chromosome count is highlighted in bold, and the most frequent count values are listed on the right. Species and count details are given in Supplementary Table S2.*

## Discussion

This study examines evidence regarding ploidy in the legume family, particularly focusing on subfamily Cercidoideae. What motivates this focus is the hypothesis that *Cercis*, sister to the remainder of the Cercidoideae, has no history of polyploidy – which may be in contrast to all other legume species. This would make *Cercis* valuable as a genomic model for the legumes, and would also help to clarify histories of chromosome evolution throughout the rest of the large and diverse legume family. Specifically, if *Cercis* did not undergo a WGD relative to the common ancestor of legumes, and if the ancestors of other lineages in the Cercidoideae, Dialioideae, Detarioideae, Caesalpinioideae, and Papilionoideae did, then the legume clade as a whole is not fundamentally polyploid relative to its sister taxa. Combined with evidence that the papilionoid WGD affects all papilionoid species but does not extend to species in the caesalpinioid or detarioid subfamilies (Cannon et al., 2015), the necessary inference is that there must have been multiple, independent events: at a minimum, one in the Cercidoideae and another in the Papilionoideae – and our findings here are also consistent with our previous conclusion of independent polyploidy events early in the Caesalpinioideae and Detarioideae (Cannon et al., 2015). We have no information about ploidy in the monogeneric Duparquetioideae; and it is not known directly whether species in the Dialioideae experienced a WGD, though chromosome counts of 12–14 in Dialioideae are consistent with the hypothesis that they too are polyploid.

The cumulative evidence that *Cercis* lacks a legume-era WGD is substantial. Recapping:

• In *K*_s_ plots (Figure 1, 2), there is no peak indicating WGD in *Cercis* – particularly, in plots derived from synteny comparisons. In contrast, such peaks are clearly evident in diverse legume lineages including *Phaseolus, Bauhinia*, and *Chamaecrista*. While there is no such peak in the *Cercis* self-comparison, there are clear peaks in comparisons of *Cercis* to each of the other species examined, indicating that the lack of *K*_s_ peak is not due to something essentially wrong with gene-calls in *Cercis* (the gene calls have homologs with the comparison legume species, and those homologs can be aligned in-frame with those homologs, giving reasonable *K*_s_ results).
• In genomic synteny comparisons between *Cercis, Phaseolus*, and *Prunus* (the latter two with known duplication histories), the duplication status of *Cercis* looks like that of *Prunus* rather than *Phaseolus* – i.e., lacking a WGD in the timeframe of the fabidae.
• In phylogenomic analyses of 14,709 gene-family trees (Table 3), sequence counts aggregated across all trees show a pattern consistent with at least one WGD in each species examined except *Cercis*. Examining the proportion of gene families with two or more sequences for a species to families with only one sequence, all species examined have a ratio ranging from 54 to 80% (and 632% for *G. max*, which had an additional recent WGD), in contrast to 24% for *Cercis*. For comparison, this ratio is 69% in the set of 177 conserved collinear genes in the triplicated *B. oleracea* genome segments identified by Town et al. (2006).
• Mining the gene families for phylogenetic topologies within the Cercidoideae (Table 4), the overwhelming majority of clades have a pattern of two *Bauhinia* sequences to one *Cercis* sequence (roughly tenfold more frequently than the other options combined).
• Diverse species within the Cercidoideae all show a pattern of duplicated *CYCLOIDEA*-family genes, with the exception of *Cercis*, which has only one *CYCLOIDEA* gene – whether assayed through amplification with degenerate primers for *CYCLOIDEA*, or through gene prediction in the *Cercis* genomic sequence (Figure 5). All phylogenetic analyses (whether based on plastid or nuclear sequences) resolve *Cercis* as sister to the remainder to Cercidoideae, in line with a WGD after the split with *Cercis* (although rooting in Figure 5 is uncertain, so *Cercis* could group with one or the other of the *CYCLOIDEA* gene forms in the gene family).
• A survey of chromosome count data for 477 legume genera, examined in a phylogenetic context (Figure 7, Table 5, Supplementary Table S4, and Supplementary Data Sheets S6, S7), shows a pattern consistent with WGDs affecting all subfamilies and most genera – with the exception of *Cercis* itself. Models in which most legumes are polyploid have been proposed in earlier studies (Goldblatt, 1981; Doyle, 2012), on the basis of chromosome numbers. In the Cercidoideae, the most frequent chromosome count is *n* = 14 for most species, but 7 in *Cercis*; in the Detarioideae, the modal chromosome count is 12; in the Dialioideae, the modal count is 14; in the Caesalpinioideae, the modal count is 14; and in the Papilionoideae, the modal count for early-diverging genera (e.g., *Swartzia, Angylocalyx, Cladrastis*), the most common counts are 13 and 14. Crown-group clades have highly variable counts (generally in the range of 7–11 chromosomes), so we hypothesize a doubling from 7 to 14 leading to the papilionoid origin, then a reduction from 14 to lower numbers for crown-group clades (dalbergioids, baphioids, mirbelioids, Robineae, Loteae, IRLC, indigoferoid, and millettioid).
• Genome sizes in the Cercidoideae are consistent with WGD in *Bauhinia* and not *Cercis*. The *Cercis* genome is approximately 367 Mbp, while values for *Bauhinia* species range from 573 to 620 Mbp. A *Cercis* genome size of 367 Mbp is tied for smallest in the legume family, and is less than a third the median reported genome size of 1,157 Mbp, across 84 legume genera. We note this result with a caveat, however, that genome sizes can be highly variable, even within a single genus – affected by mechanisms such as bursts of transposon expansions – e.g., variations in *Nicotiana* (Leitch et al., 2008) or in *Aeschynomene* (Brottier et al., 2018).

Further analyses of evolutionary changes due to the differing WGD status between *Cercis* and other legumes will be of interest – both at the fine scale (e.g., determining the fate of duplicated genes in various lineages, relative to *Cercis*) and at larger structural scales (e.g., determining structural changes in chromosomes following several independent WGD events) These comparisons would benefit from improved assemblies and annotations, spanning a broader range of legume clades. For example, we expect both *Chamaecrista* (as a nodulator in the Mimosoideae) and *Cercis* (as an early-diverging non-nodulator) to be useful in better understanding the origin and evolution of nodulation symbioses – as investigated in several recent papers (Battenberg et al., 2018; Griesmann et al., 2018; van Velzen et al., 2018).

An initially puzzling result from our analysis was the fact that the *K*_s_ peak for the *Bauhinia* self-comparison (*Bauhinia–Bauhinia*) appears significantly “older” than the *Bauhinia–Cercis* speciation peak, at 0.25 and 0.15, respectively (Figure 1A). Similarly, most gene tree topologies (63%) that have two or more *Bauhinia* sequences and one *Cercis* sequence (Table 4, row 3) have a configuration of (B,(B,C)), indicating duplication prior to speciation – in contrast to what might be expected given a simple model of *Cercis–Bauhinia* speciation followed by WGD in *Bauhinia*. In the latter case, the expected pattern would be [(B,B),C] – which is observed in the minority of cases (37%). We note that an apparent speciation pattern may be due either to a WGD or to local, private duplications. Private duplications are common in plant genomes. For example, in *M. truncatula*, more than a third of paralogs are derived from local duplications (Young et al., 2011). However, local duplications tend to be evident in *K*_s_ plots as a recent peak, with maximum near zero – as is seen, for example, in the *Phaseolus–Phaseolus* comparison in Figure 1. This is the typical pattern described by Lynch and Conery (2000) for eukaryotes generally. The results of our phylogenetic pattern-mining tests are consistent with what we observe (albeit anecdotally) in visual inspection of many trees, exemplified by Figure 3, in which there is a duplication of the *Bauhinia* paralogs in both trees, apparently followed by orthologous split between one of the *Bauhinia* sequences and the *Cercis* sequence.

**FIGURE 8:**
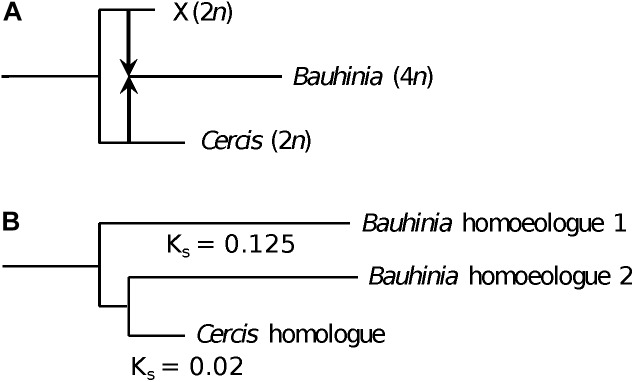
Allopolyploid origin of *Bauhinia*. **(A)** Species history, showing divergence between two diploid (2*n*) species: (1) the ancestor of *Cercis* and (2) a second species that became extinct (“X”). At some point after the species divergence, the two diploid species hybridized (arrows), followed by genome doubling to produce the allopolyploid (4*n*) ancestor of *Bauhinia* (and other Cercidoideae). **(B)** Representative gene tree sampled from *Bauhinia* and *Cercis*, showing the relationships of the single homologous gene in *Cercis* to the two homoeologs in allopolyploid *Bauhinia*. The *Bauhinia* homoeolog 2, contributed by the *Cercis* ancestor, is sister to the *Cercis* gene. The Cercis gene has a *K*_s_ of ∼0.145 compared with the *Bauhinia* homeolog 2; and each *Bauhinia* homoeolog has a *K*_s_ of 0.25 with respect to the other *Bauhinia* homoeolog. The relationship between the species history and the gene tree is complicated by the hypothesized slower substitution rate in Cercis.

A model that could accommodate the *K*_s_ and tree-topology results is one of allopolyploidy, in which a progenitor of *Cercis* speciated to give another (perhaps now-extinct) diploid species (Figure 8A). These species diverged for some time, and then the two species contributed their genomes to a new allopolyploid species that was the progenitor of the remaining Cercidoideae. Following allopolyploidy, the two lineages (diploid *Cercis* and polyploid *Bauhinia*) would then have proceeded to diverge and diversify – *Cercis* more slowly and the remaining species in Cercidoideae more rapidly. The current gene family view would then be as observed in e.g., Figure 3, or in the model in Figure 8B.

Precedent for a significant period of species divergence prior to allopolyploidy is seen, for example, in *Arachis*: the allopolyploid *A. hypogaea* was formed, within about the last 10 thousand years, from the merger of *A. duranensis* and *A. ipaensis*, which diverged an estimated 2.16 Mya (Bertioli et al., 2016). Another similar example is in cotton, where the allotetraploid *Gossypium hirsutum* L. is a merger of genomes from progenitor species similar to the extant diploid species *G. ramondii* Ulbrich and *G. herbaceum* L. (Wendel, 1989; Flagel et al., 2012; Paterson et al., 2012) In this case, the diploid species diverged c. 5–10 Mya and merged to form *G. hirsutum* c. 1–2 Mya (Wendel, 1989; Fang et al., 2017).

The genus *Cercis* contains 10 species and all phylogenetic analyses to date have supported the genus as monophyletic. This is a well-defined group of north temperate trees (North America, Eurasia and eastern Asia). All species for which counts are available are diploid^2^. There appears to be relatively low genetic diversity within the genus based on plastid and nuclear ribosomal ITS sequences (Davis et al., 2002; Coskun and Parks, 2009). *C. chingii* (*n* = 14) is resolved as sister to the other species in the genus in the studies by Davis et al. (2002), and differs from the other species by its coriaceous, unwinged, dehiscent fruit. The other species are morphologically quite similar. It’s not clear if one of the present day *Cercis* species could better represent an ancestral parental genome resulting in the whole genome duplication.

*Cercis* genes do appear to have evolved remarkably slowly (at least in the sense of accumulating point mutations that affect *K*_s_ and branch lengths). A tree calculated by algebraically solving evolutionary “distance paths” along a gene tree (Figure 1, 2, lower right), using *K*_s_-based branch lengths, shows a *Cercis* evolutionary rate less than a quarter that of *Bauhinia*, and roughly a tenth that of *Phaseolus* since the papilionoid WGD. The slow *Cercis* rate is also evident in many gene family trees, such as the two shown in Figure 3. The *matK* gene tree also shows remarkably short branches for *Cercis*. It is conceivable that the slower evolutionary rate seen in *Cercis* than other legumes might be partly due to the lack of WGD-derived “extra” genes in *Cercis* –perhaps presenting extra evolutionary constraints than for duplicated genes. The outcrossing, long-lived tree form might also constrain evolutionary rates (injecting older gametes into new progeny) – although of course these conditions are shared with many species.

## Conclusion

The evidence from diverse sources indicates that *Cercis* may be unique among legume lineages in lacking any evidence for a WGD; that its last duplication event was probably the eudicot “gamma” triplication event; that the genomes of other Cercidoideae and all other legume subfamilies are likely to have been shaped by independent WGD events; that the most likely model for WGD and speciation timing in the Cercidoideae is allopolyploidy – with a *Cercis* progenitor contributing one subgenome to the allopolyploid *Bauhinia* progenitor; and lastly, that *Cercis* has evolved at a strikingly low rate since its divergence from other Cercidoideae. Taken together, these findings suggest that *Cercis* may serve as a useful genomic model for the legumes, likely representing the duplication status of the progenitor of all legumes.

## Author Contributions

SC, JD, and DF-B conceptualized the research. JS, AY, SC, and DF-B planned and constructed gene families. JS and AY conducted phylogenomic tests. AB and CS generated and assembled *CYCLOIDEA* sequences for species phylogenetic analyses. SC, JS, and AY drafted the manuscript. All authors reviewed and contributed to the manuscript.

## Conflict of Interest Statement

The authors declare that the research was conducted in the absence of any commercial or financial relationships that could be construed as a potential conflict of interest.

## Footnotes

1 https://github.com/akshayayadav/clade-based-family-analysis

2 http://www.tropicos.org/Project/IPCN

## Supplementary Material

TABLE S1 Histograms of *K*_s_ values for selected species pairs, calculated using top BLAST matches and calculated using gene pairs from genomic synteny features.

TABLE S2 Counts of species in legume gene families.

TABLE S3 Sequence IDs and accessions for *CYCLOIDEA*-like genes from species in the Cercidoideae.

TABLE S4 Sequences for legume matK genes, and chromosome counts for legume species, with modal chromosome counts per genus.

TABLE S5 Legume genome size estimates.

DATA SHEET S1 legume.genefam.esm.RDQM.SD01_65_hmmalign Full alignments of all legume gene families that contain *Cercis* and/or *Bauhinia*.

DATA SHEET S2 legume.genefam.esm.RDQM.SD02_65_hmmalign_trim2 Alignments of all legume gene families that contain *Cercis* and/or *Bauhinia*, trimmed to HMM “match states,” prior to calculation of phylogenetic trees.

DATA SHEET S3 SD03_70_trees_combined Phylogenetic trees, calculated using RAxML, and rooted by the nearest non-legume outgroup species.

DATA SHEET S4 SD04_71_trees_reduced_trees Phylogenetic trees as in SD03 but with same-species terminal branches reduced to a single sequence representative, for terminals with branch lengths < 0.2.

DATA SHEET S5 SD05_tree_legcyc_and_outgrps7boot_color.nh.txt Phylogenetic tree, calculated using RAxML, for *CYCLOIDEA*-like genes from species in the Cercidoideae.

DATA SHEET S6 Phylogenetic tree image for all legume genera with chromosome counts, with modal chromosome counts per genus, colored by subfamily.

DATA SHEET S7 SD11_LPWG_chrom_counts_by_genus3.tree.txt Phylogenetic tree file, in phylip format, corresponding with the image in SD10.

DATA SHEET S8 Synteny plots between *Cercis canadensis, Phaseolus vulgaris*, and *Prunus persica*.

DATA SHEET S9 SD09_quota_tables Results from quota-alignment pipeline for comparisons between *C. canadensis, Phaseolus vulgaris*, and *Prunus persica*.
