# Supplementary figures and images for: Cercis: A Non-polyploid Genomic Relic Within the Generally Polyploid Legume Family
Source: Front Plant Sci. 2019 Apr 11;10:345. doi: 10.3389/fpls.2019.00345 (PMC6499179; doi:10.3389/fpls.2019.00345)

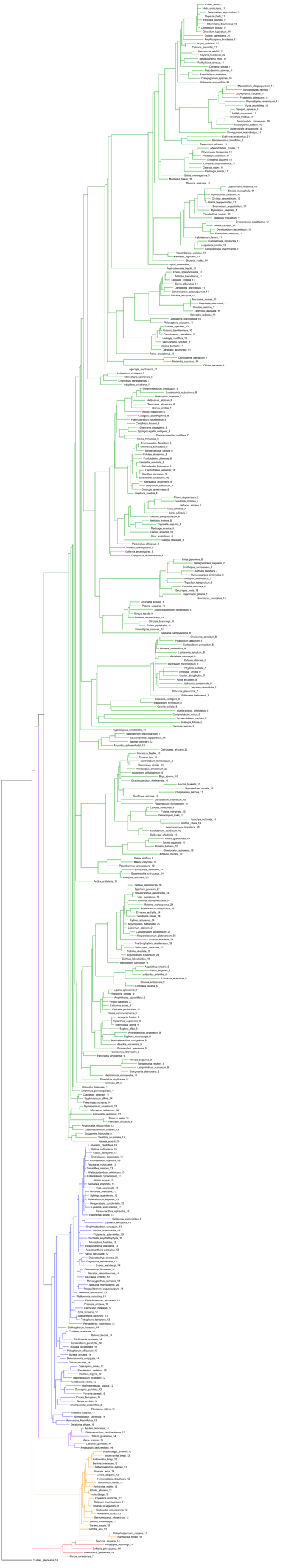

Supplement: DATA SHEET S6 — Phylogenetic tree image for all legume genera with chromosome counts, with modal chromosome counts per genus, colored by subfamily. [file Data_Sheet_6.pdf]

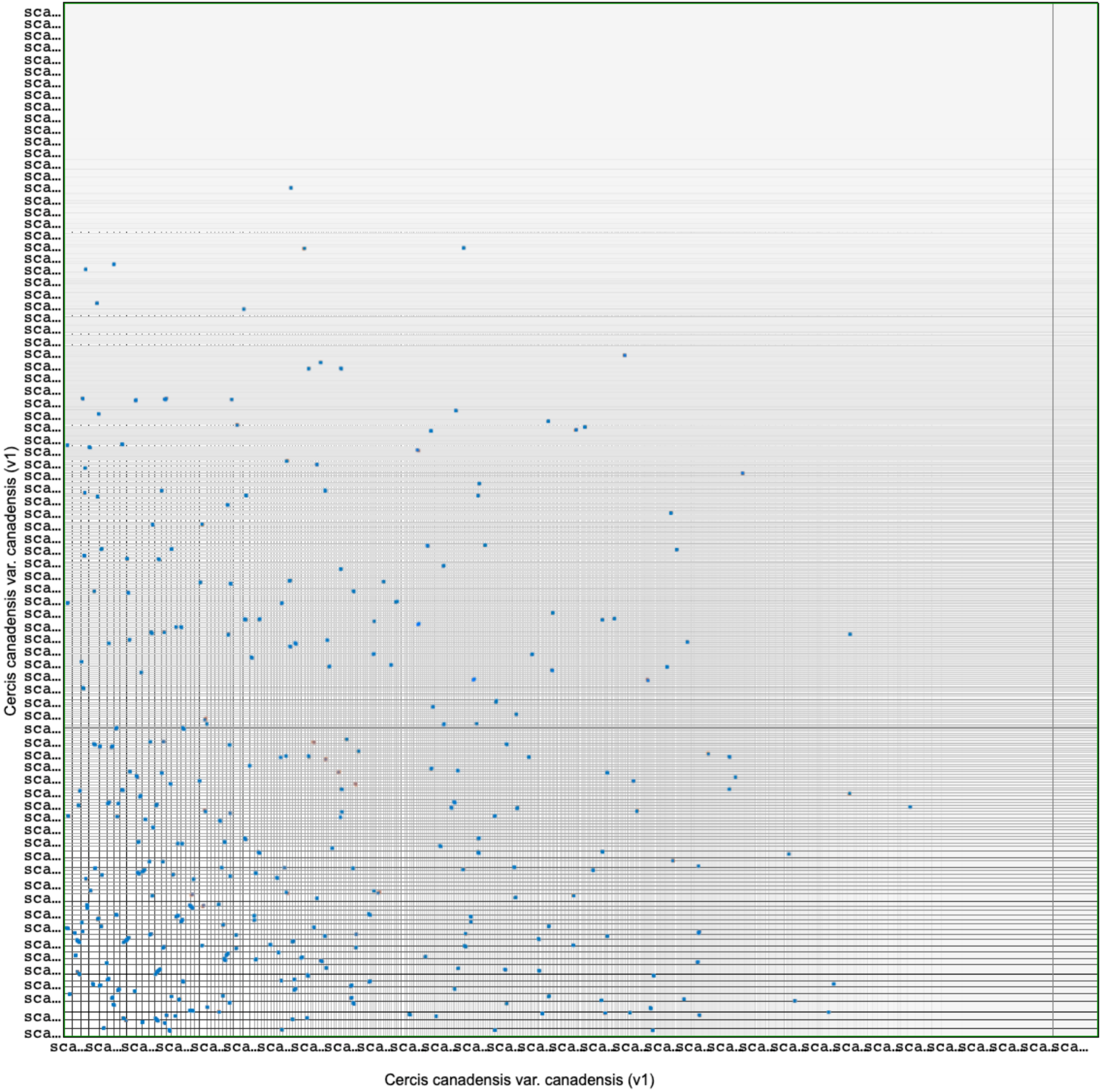

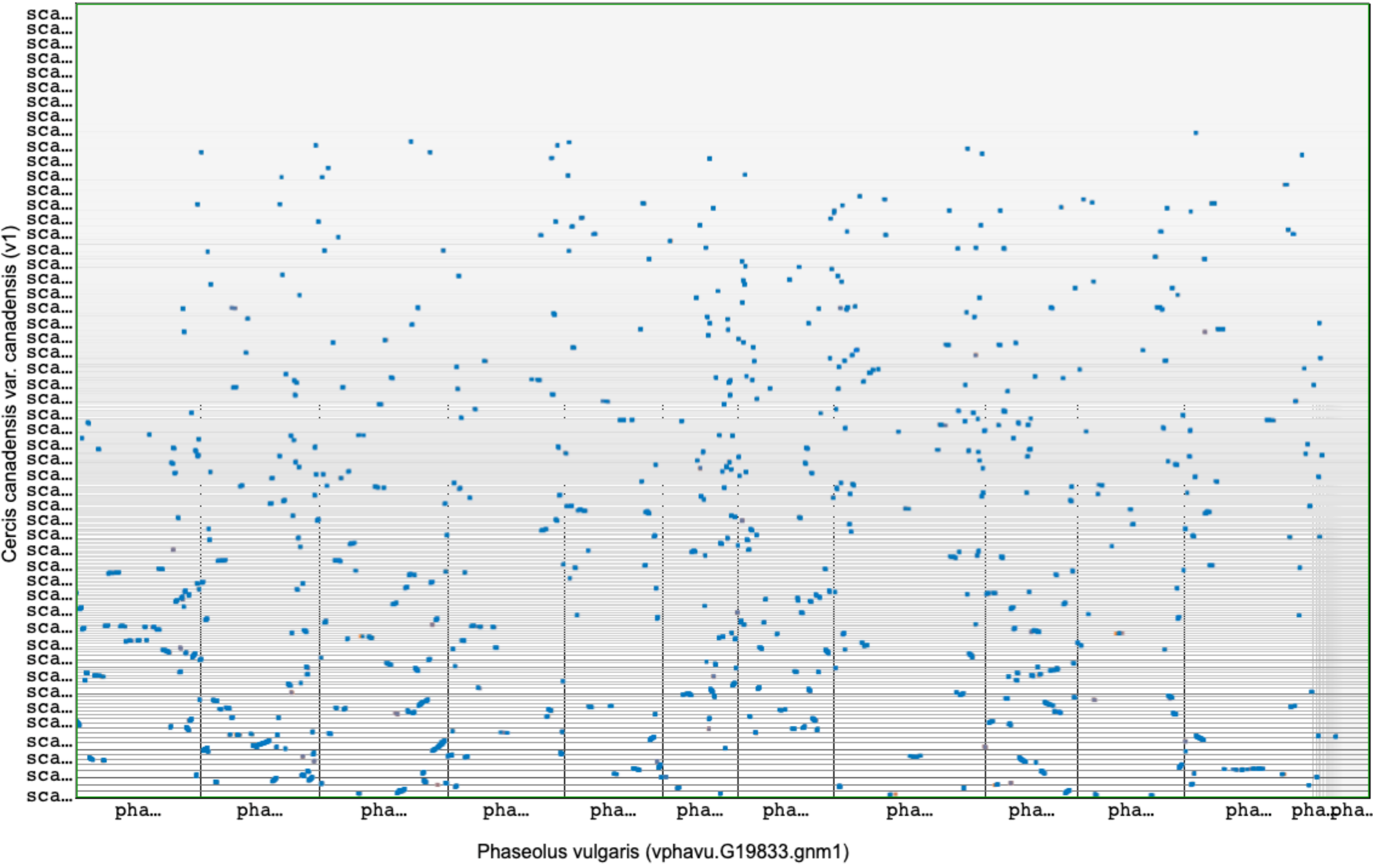

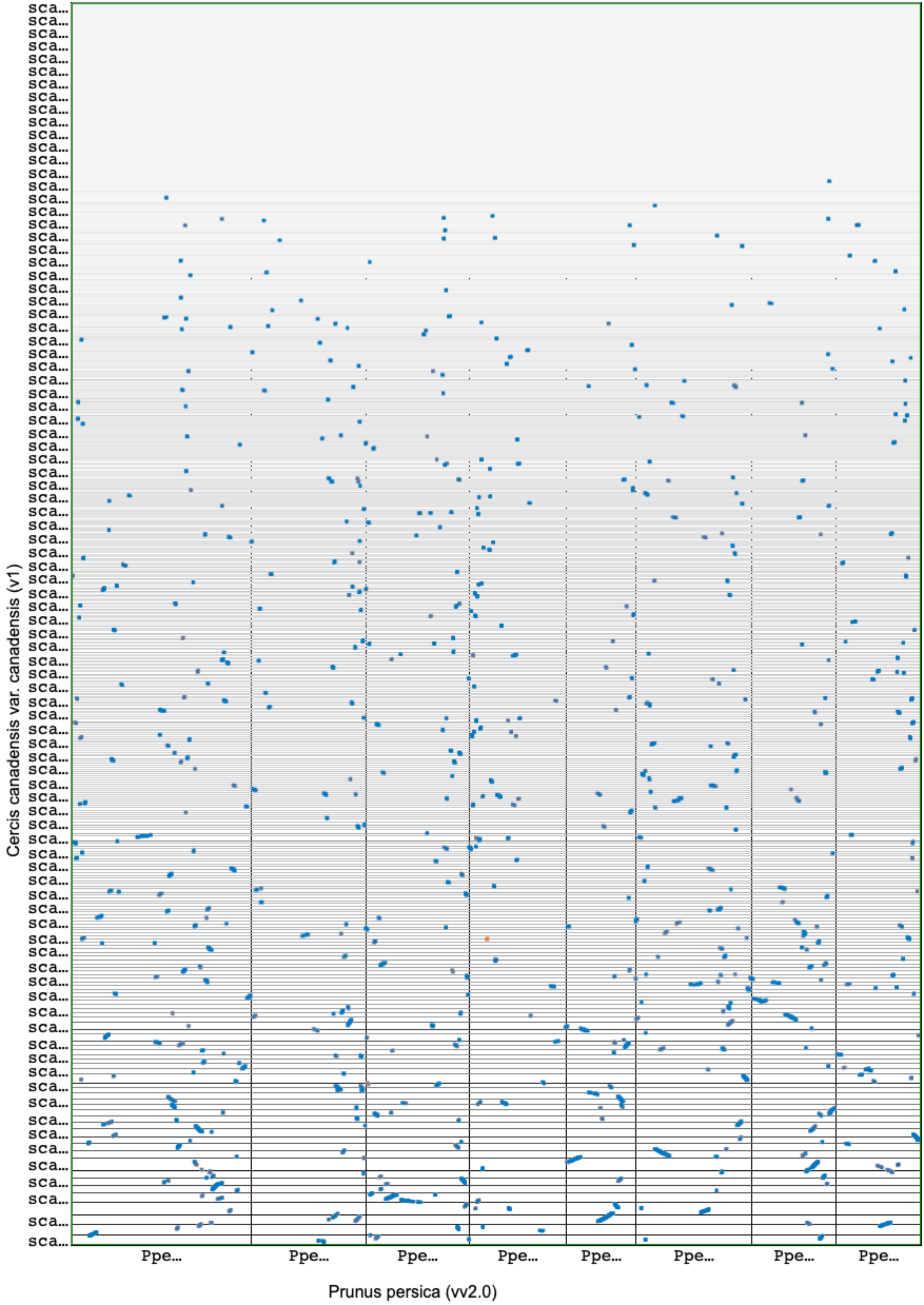

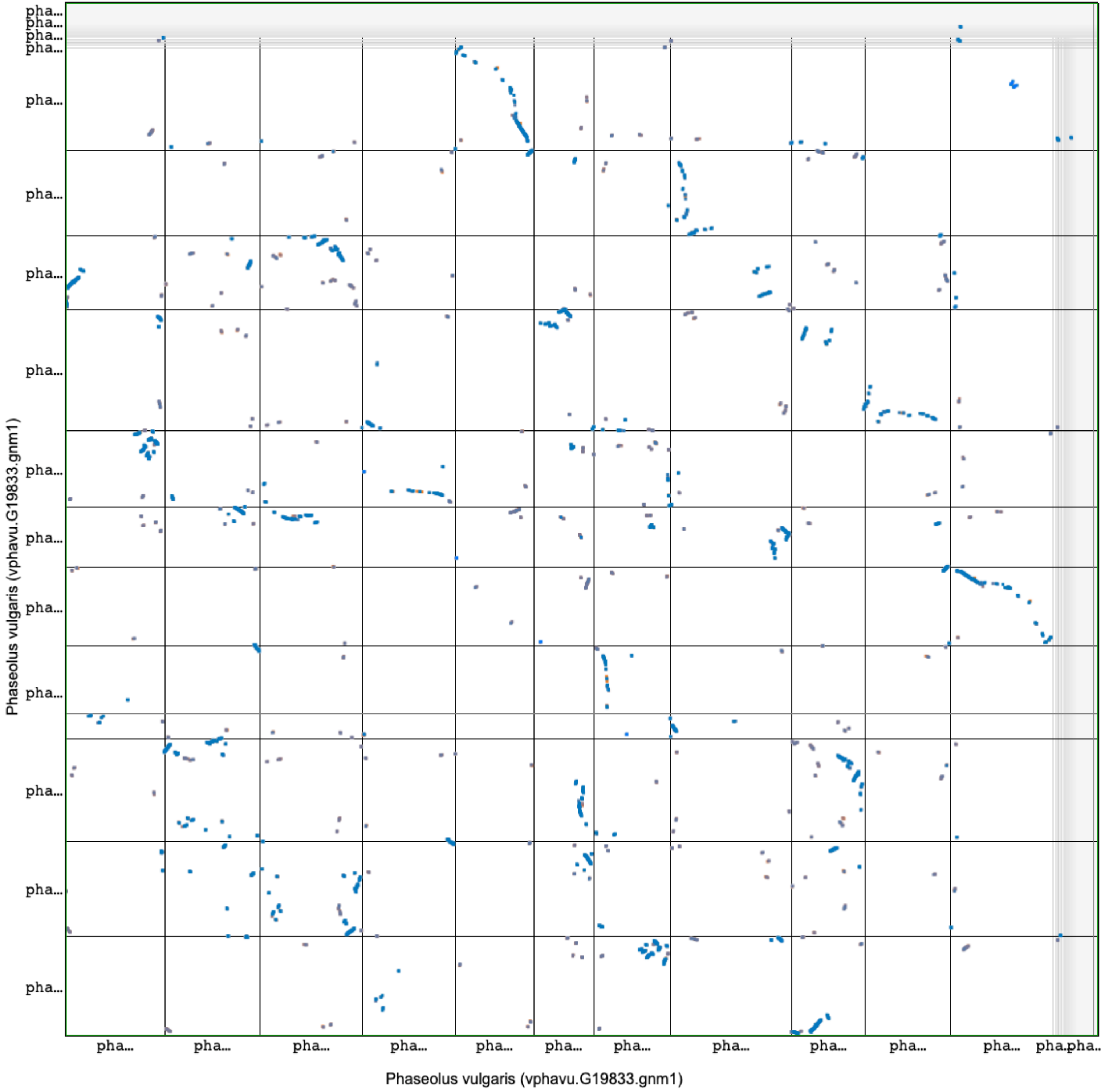

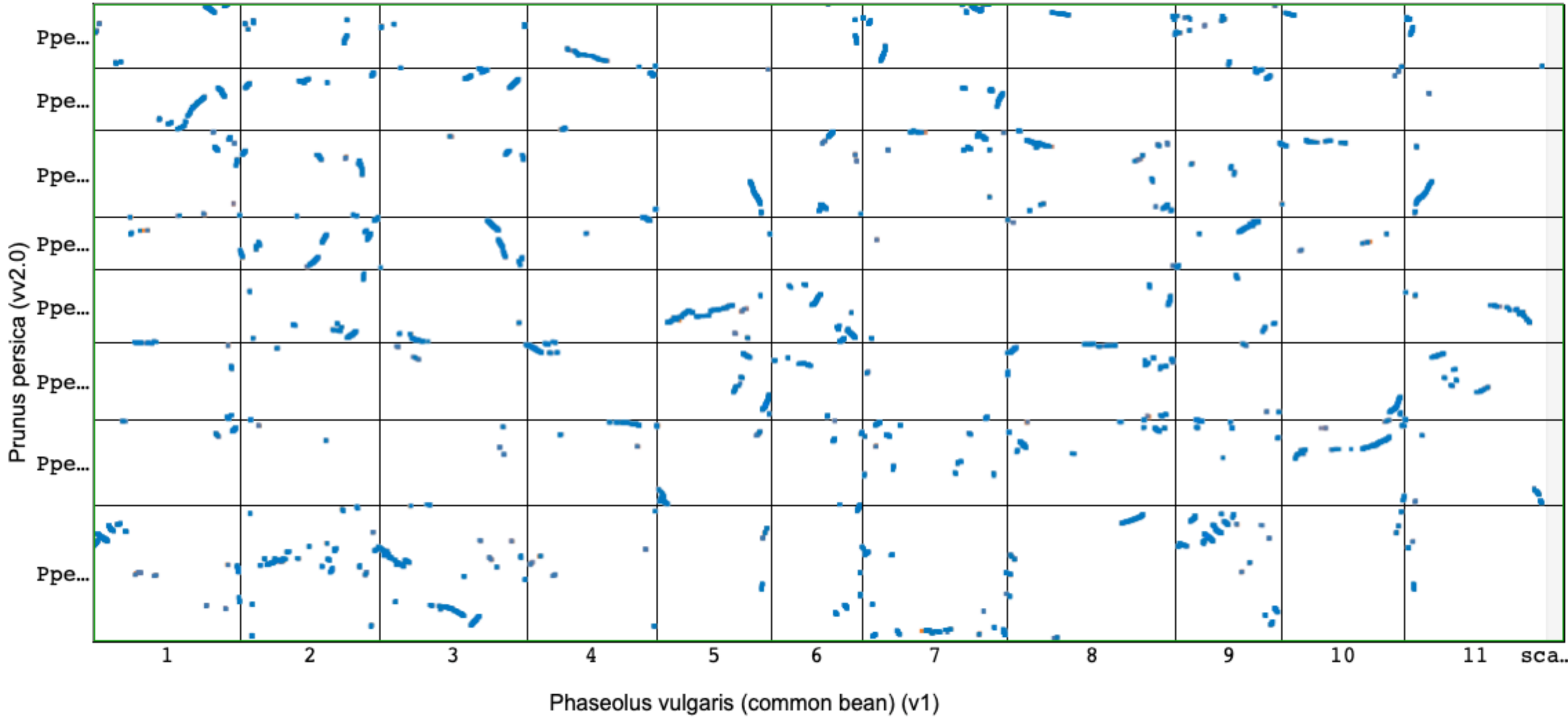

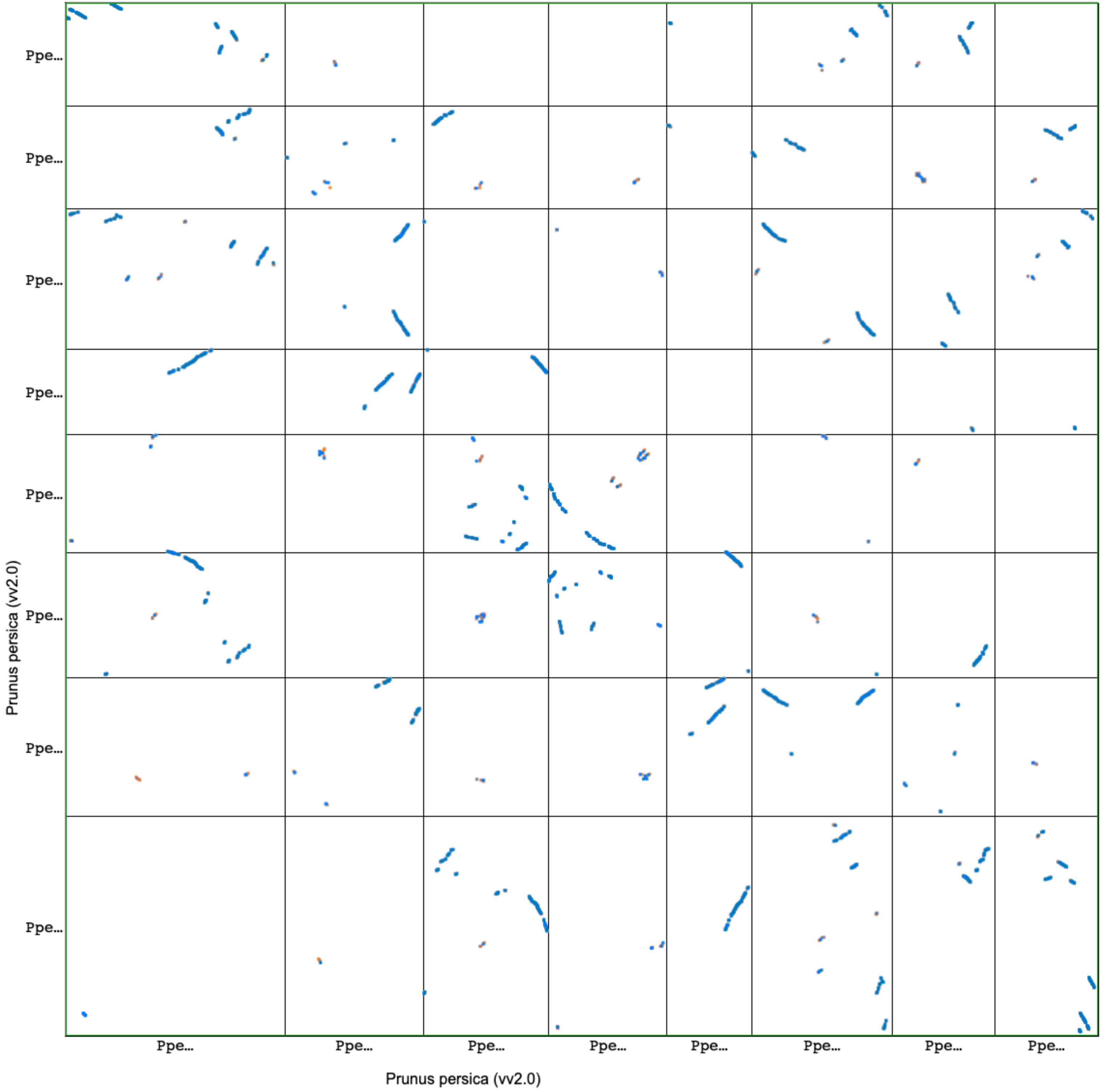

Supplement: DATA SHEET S8 — Synteny plots between Cercis canadensis, Phaseolus vulgaris, and Prunus persica. [file Data_Sheet_8.pdf]
